# Hydroxyapatite Particle Density Regulates Osteoblastic Differentiation Through β-Catenin Translocation

**DOI:** 10.3389/fbioe.2020.591084

**Published:** 2021-01-08

**Authors:** Otto J. Juhl, Anna-Blessing Merife, Yue Zhang, Christopher A. Lemmon, Henry J. Donahue

**Affiliations:** Department of Biomedical Engineering and Institute for Engineering and Medicine, Virginia Commonwealth University, Richmond, VA, United States

**Keywords:** mechanotransduction, focal adhesion, surface topography, translocation, beta catenin

## Abstract

Substrate surface characteristics such as roughness, wettability and particle density are well-known contributors of a substrate's overall osteogenic potential. These characteristics are known to regulate cell mechanics as well as induce changes in cell stiffness, cell adhesions, and cytoskeletal structure. Pro-osteogenic particles, such as hydroxyapatite, are often incorporated into a substrate to enhance the substrates osteogenic potential. However, it is unknown which substrate characteristic is the key regulator of osteogenesis. This is partly due to the lack of understanding of how these substrate surface characteristics are transduced by cells. In this study substrates composed of polycaprolactone (PCL) and carbonated hydroxyapatite particles (HAp) were synthesized. HAp concentration was varied, and a range of surface characteristics created. The effect of each substrate characteristic on osteoblastic differentiation was then examined. We found that, of the characteristics examined, only HAp density, and indeed a specific density (85 particles/cm^2^), significantly increased osteoblastic differentiation. Further, an increase in focal adhesion maturation and turnover was observed in cells cultured on this substrate. Moreover, β-catenin translocation from the membrane bound cell fraction to the nucleus was more rapid in cells on the 85 particle/cm^2^ substrate compared to cells on tissue culture polystyrene. Together, these data suggest that particle density is one pivotal factor in determining a substrates overall osteogenic potential. Additionally, the observed increase in osteoblastic differentiation is a at least partly the result of β-catenin translocation and transcriptional activity suggesting a β-catenin mediated mechanism by which substrate surface characteristics are transduced.

## Introduction

Studies evaluating the effect of substrate surface characteristics on osteogenesis have determined that there is a significant interplay between surface characteristics and bone cell response (Lim et al., 2011; Abagnale et al., 2015). For instance, recent studies have demonstrated that micro and nano-scale topographies, by affecting surface characteristics such as roughness, wettability, and height, effect changes in the cell's cytoskeletal structure (Lossdörfer et al., 2004; Lim et al., 2005c; Yim et al., 2010). These topographies also promote osteogenesis and direct mesenchymal stem cells toward the osteoblastic lineage (Deligianni et al., 2000; Mendonça et al., 2009). Frequently, the substrate surface characteristics are created by incorporating a pro-osteogenic material, such as hydroxyapatite particles (HAp), directly into the substrate.

Hydroxyapatite is the main mineral component of bone and is highly osteoconductive. HAp are synthesized in a variety of sizes, from the nanometer to micron scale, and a variety of shapes, from spheres to needle-like rods (Roohani-Esfahani et al., 2010; Tautzenberger et al., 2012). The effect of various HAp sizes and shapes on their osteogenic potential has been summarized in numerous reviews that suggest spherical HAp <1 micron in size facilitate osteogenesis to the greatest degree (Legeros et al., 1967; Roohani-Esfahani et al., 2010; Tautzenberger et al., 2012). Unfortunately, hydroxyapatite is not a viable material for all load bearing applications because of its extreme brittleness during shear (Roohani-Esfahani et al., 2010; Gordeladze et al., 2017). To overcome this, HAp are typically combined with other materials, including polymers such as polycaprolactone (PCL), which is biocompatible, bioresorbable, and exhibits mechanical properties more similar to that of bone (Gordeladze et al., 2017).

Combining HAp and PCL has resulted in materials that have unique and tunable surface characteristics. By increasing or decreasing the ratio of HAp to PCL, variations in substrate surface characteristics such as roughness, particle density, wettability, and height can be achieved (Thomas et al., 2006; Wu et al., 2015; Bovand et al., 2019). Studies investigating these substrate properties have yielded conflicting results because the substrates examined have numerous properties that differ. This has made identifying a singular property that is causing osteogenesis challenging. To overcome this, previous studies have cultured cells on nanoposts, limiting the differences in substrate characteristics and instead varying only post-size, post-height, and post-density. These studies determined that the presentation of denser nanoposts to human mesenchymal stem cells resulted in more adipogenic differentiation, while less dense substrates resulted in a more osteogenic differentiation (Ahn et al., 2014). Moreover, nanoposts that were between 200 and 700 nm in diameter were the most osteogenic, but this was also dependent on post-height and stiffness (Wen Kuo et al., 2014). These studies suggest that the ability of the cell to form the correct cell-substrate adhesion, known as a focal adhesion, is a significant contributor to inducing osteogenesis. However, translation of these results to materials that can be utilized *in vivo* such as PCL and HAp have yielded conflicting results.

One reason it is still challenging to create novel substrates with increased osteogenic potential is because it is still unknown how substrate surface characteristics are transduced and how this signal then regulates osteogenesis (Pavalko et al., 2003; Kashte et al., 2017). Numerous attempts have been made to determine the mechanism by which substrate surface characteristics are transduced into intracellular signals. This has given rise to the concept that surface characteristics induce cytoskeletal changes that then alter nuclear morphology and gene expression (Schaffler and Kennedy, 2012). Previous data also suggest β-catenin, a protein found at cell adhesions and a key contributor in the Wnt signaling pathway, transduces substrate surface characteristics, but this concept has yet to be validated (Alenghat and Ingber, 2002; Perez-Moreno et al., 2003).

The canonical Wnt signaling pathway relies on β-catenin translocation to the nucleus to regulate transcription factors, which in the case of osteoblastic cells, regulate pro-osteogenic genes (Cadigan and Waterman, 2012). Another role of β-catenin is its contribution to the formation and stabilization of cell adhesions such as focal adhesions and cadherins (Mbalaviele et al., 2006; Thompson et al., 2012). At E-cadherins, β-catenin binds the cadherin directly at the N-terminus, where it is then stabilized by α-catenin. α-catenin then either binds directly to actin or indirectly to vinculin that then binds actin (Jamora and Fuchs, 2002). More recent studies have also suggested that vinculin may directly bind β-catenin after activation and that the β-catenin/vinculin complex is capable of supporting mechanical tension (Peng et al., 2010; Bertocchi et al., 2019). Interestingly, vinculin is also a component of another adhesion complex, focal adhesions, although the role of β-catenin, if any, at focal adhesions is still unknown (Kanchanawong et al., 2010).

Focal adhesion complexes are composed of >50 proteins and are known to contribute to mechanosensing within the cell (Zamir and Geiger, 2001). The transmembrane portion is comprised of integrins that bind to the extracellular matrix. Previous studies suggest that integrins respond differentially to various surface characteristics, with different forms of integrins adhering preferentially to pro-osteogenic substrates compared to sub-optimal substrates (Hyzy et al., 2017). Focal adhesions, well-known mechanosensors, also increase in size in response to increased actin fiber tension. This phenomena, known as the growth model of force-induced focal adhesion, is driven by actomyosin-mediated tension (Besser and Safran, 2006; Geiger et al., 2009; Kim and Wirtz, 2013; Kuo, 2014). Previous studies examining human mesenchymal stem cell differentiation have observed a correlation between nanopost density, focal adhesion formation and maturation, and the differentiation state of the cell (Di Cio and Gautrot, 2016). These studies found the median densities often elicit the greatest increase in focal adhesion maturation and differentiation. In addition, evidence presented by Dubrovskyi et al. suggests that β-catenin may localize to focal adhesions as well, binding paxillin during Rac activation (Dubrovskyi et al., 2013). However, the contribution β-catenin may have facilitating focal adhesion formation and binding of actin stress fibers has not been fully explored. The relationship between focal adhesion maturation, osteoblastic differentiation, and β-catenin localization has led to the examination of whether focal adhesions transduce substrate surface characteristics to mediate osteogenesis and, if so, the mechanism by which this occurs (Perez-Moreno et al., 2003; Nelson and Nusse, 2004; Marie et al., 2014).

To better understand the mechanism by which substrate surface characteristics affect osteogenesis we varied the concentration of HAp independent of PCL to create substrates with a range of surface characteristics. We hypothesized that substrates with a concentration of 30% HAp to PCL would increase osteoblastic differentiation compared to lower (5%) or higher (50%) concentrations of HAp to PCL, similar to that which has been observed in previous studies using various densities of nanoposts (Di Cio and Gautrot, 2016). Furthermore, the mechanism by which this occurs may involve the regulation of β-catenin by, or liberation from, proteins at adhesion complexes, located at the cell membrane. This would result in increased β-catenin translocation form these adhesion complexes to the nucleus on the more osteogenic substrate.

To evaluate this hypothesis, we examined osteoblastic differentiation, as assessed by osteoblastic gene expression and alkaline phosphatase (AP) activity, on 5, 30, and 50% HAp to PCL substrates. We also evaluated focal adhesion morphology and number over time, β-catenin localization over time, and nuclear β-catenin activity. We observed that cells on substrates with a concentration of 30% HAp to PCL displayed increased expression of genes associated with osteoblastic differentiation and osteoblastic activity, relative to cells on other substrates examined. However, the substrates examined did not exhibit significant changes in surface roughness, wettability, or height. Moreover, focal adhesion turnover and maturation of cells on the substrate with a concentration of 30% HAp to PCL occurs more rapidly compared to cells on tissue culture polystyrene (TCPS). This more rapid turnover and maturation also corresponded with a more rapid release of β-catenin from the membrane bound fraction, where β-catenin is retained at adherens junctions and focal adhesions, to the nucleus.

## Materials and Methods

### Substrate Synthesis and Characterization

#### Polycaprolactone and Hydroxyapatite Substrate Synthesis

To create a pro-osteogenic substrate to test our hypothesis, various formulations of HAp and PCL substrates were fabricated. Five thousand molecular weight PCL was suspended at a 2.5% wt./vol. ratio in chloroform. The mixture was then homogenized until all PCL was dissolved into solution. After homogenization, 500 nm HAp was added into the solution at a ratio of 5, 30, or 50% wt./wt. with the PCL. Using a dip-coating technique, 22 mm coverslips were coated with one of the following; PCL solution only (PCL), 5% HAp to PCL solutions, 30% HAp to PCL solutions, or 50% HAp to PCL solution. After coating, the various substrates were allowed to dry at room temperature for 24 h.

#### Substrate Surface Roughness and Height Characterization

Substrate surfaces were characterized for roughness and height (z-range) using a Dimension ICON atomic force microscopy platform (Veeco, Plainview, NY). Briefly, each substrate was rinsed with 70% ethanol to remove debris left on the surface after synthesis and the ethanol allowed to evaporate. The substrates where them imaged by atomic force microscopy using a TESP-SS antimony doped silicon cantilever (Bruker, Billerica, MA). The cantilever used a single tip with a resonance frequency in the range of 230–410 KHz. The probe radius was 5 nm, with a tip height of 10–15 μm, spring constant of 42 N/m, and a cone angle of <10° over the first 200 nm of tip length. All evaluations were performed in air. The set point voltage was kept between 0.1 and 0.3 V with the cantilever force kept constant. The frequency employed was manipulated based on the substrate in order to accurately quantify the surface roughness and height. The scan resolution was 256 × 256 pixels with a scan frequency of 0.85 Hz over a 5 μm × 5 μm area scanned. The average height and roughness of three random areas per substrate was determined and used to estimate each substrate's overall roughness and height.

#### Substrate Surface Energy Characterization

Surface energy, also referred to as wettability, was characterized using contact angle measurements with a 590 Advanced Automated Goniometer/Tensiometer (Ramé-Hart, Succasunna, NJ). Briefly, each substrate was rinsed with 70% ethanol to remove any debris left on the surface after synthesis and the ethanol allowed to evaporate. The substrates then underwent contact angle measurement using doubly distilled water. Three random areas were evaluated on each substrate and the average of the three measurements was taken as the substrate's overall wettability. Image analysis was performed by using the DROPimage version 2.4 software (Ramé-Hart, Succasunna, NJ).

#### Substrate Particle Density Characterization

Particle density for the various substrates was quantified using an EVOS light microscope. Images of the substrate were taken at three random locations. Using Fiji image software (Schindelin et al., 2012), HAp was isolated by increasing the contrast between the HAp, which auto-fluoresces, and the background image using a 0.3% increase in saturated pixel contrast. The images were then converted to an 8-bit file format and then the background image subtracted using a 5-pixel rolling ball radius leaving only HAp visible. The particles were then outlined before being quantified using the Analyze Particles tool package in Fiji (Schindelin et al., 2012). The average particle number of the three random locations was then determined and divided by the field of view to determine the substrates areal particle density (particles/cm^2^).

### Mechanistic Evaluation of Substrates

#### Osteoblast Cell Culture

To assess osteogenesis, the differentiation capacity of human fetal osteoblasts (hFOB 1.19 cells), a preosteoblastic cell line, was assessed as previously described (Lim and Donahue, 2007). Briefly, for proliferation of hFOB 1.19 cells, cells were cultured at 33.5°C with 5% CO_2_ to 80% confluence in standard DMEM:F12 media supplemented with 10% fetal bovine serum (Gibco, Gaithersburg, MD) and 1% Penicillin/Streptomycin mixture (Sigma, St. Louis, MO). To induce differentiation of the hFOB 1.19 cells, the standard media was supplemented with 100 μg/mL ascorbic acid, 10^−8^ M menadione, and 10^−8^ M dihydroxy-vitamin D3 (Sigma, St. Louis, MO), and the cells were cultured at 39.5°C with 5% CO_2_ until the desired timepoint. Media was changed every 3 days unless otherwise stated for all experimental methods. All substrates used were sterilized by completely submerging the substrates in 70% ethanol for 5 min before the ethanol was aspirated and remaining ethanol allowed to evaporate for 1 h under UV light.

#### Osteoblastic Gene Expression

hFOB 1.19 cells were cultured on tissue culture polystyrene (TCPS), PCL, 5% HA/PCL substrate, 30% HA/PCL substrate, or 50% HA/PCL substrate and cultured under differentiation conditions for 1 and 7 days. At each time point RNA was isolated using an RNeasy Mini Kit (Qiagen, Hilden, Germany). Quantitative real time PCR (RT-qPCR) was performed using a C1000 Touch Thermal Cycler with CFX96 Real-Time System (Bio-Rad Laboratories, Hercules, CA). PowerUp Sybr Green Master Mix was used to quantify gene expression (Thermo Fisher Scientific). The genes we evaluated are associated with the various stages of osteoblastic differentiation and are as follows; alkaline phosphatase (Alpl), Osteocalcin (Bglap), Collagen 1a1 (Col1-a1), RUNX Family Transcription Factor 2 (Runx2), and Sp7 Transcription Factor (Sp7). Glyceraldehyde-3-Phosphate Dehydrogenase (Gapdh) was used as a reference gene for all samples. All primers used in this study were PrimePCR Sybr Green Assay Primers (Bio-Rad Laboratories, Hercules, CA) and the unique assay ID numbers are provided in Table 1. The ΔΔCt method used to quantify fold-change in gene expression relative to the housekeeping gene Gapdh, as previously described (Tarkkonen et al., 2017).

**Table 1:** Reference numbers associated with each osteoblastic gene evaluated using primers purchased from.

**Gene of interest**	**Gene abbreviation**	**Primer reference number**
Alkaline Phosphatase	Alpl	qHsaCID0010031
Osteocalcin	Bglap	qHsaCED0038437
Collagen1-a1	Col1-a1	qHsaCEP0050510
RUNX Family Transcription Factor 2	Runx2	qHsaCID0006726
Sp7 Transcription Factor	Sp7	qHsaCEP0025867
Glyceraldehyde-3-Phosphate Dehydrogenase	Gapdh	qHsaCEP0041396

#### Alkaline Phosphatase (AP) Activity

hFOB 1.19 cells were cultured on either tissue culture polystyrene (TCPS), PCL substrate, 5% HA/PCL substrate, 30% HA/PCL substrate, or 50% HA/PCL substrate at 15,000 cells/cm and cultured under differentiation conditions for 7 days. Differentiation was evaluated using a colorimetric AP enzymatic activity assay as previously described (Donahue et al., 2000). Briefly, cells were freeze thawed in 400 μL of 0.05% Trition-X100 in phosphate buffered saline twice and then the cell lysate collected. Ten microliter of each sample lysate was removed and used to quantify total protein concentration using Pierce BCA protein assay kit (Thermo Fisher, Waltham, MA). AP enzymatic activity was then determined by conversion of *p*-nitrophenyl phosphate to *p*-nitrophenol. Two hundred microliter of AP reaction buffer was then added to each sample and incubated at room temperature for 30 min. After incubation 50 μL of each sample was moved to 200 μL of 0.1 NaOH in a 96 well-plate to quench the reaction. All samples were then measured at 410 nm and SIGMA units calculated based on the standard curve. All readings were then normalized to total protein concentration to control for variations in cell number between samples.

#### Focal Adhesion Staining

hFOB 1.19 cells were cultured on either glass, PCL, 5% HA/PCL substrate, 30% HA/PCL substrate, or 50% HA/PCL substrate at 15,000 cells/cm and cultured under differentiation conditions for 1, 2, 4, 12, 24, or 48 h. At the selected time point, cells were stained using a FAK 100 Actin Cytoskeleton and Focal Adhesion Staining Kit (Sigma, St. Louis, MO). Briefly, cell culture medium was removed, and cells were fixed in 4% paraformaldehyde for 15 min at room temperature. After fixation, cells were washed twice with wash buffer consisting of 0.01% Tween-20 in PBS. Cells were then permeabilized with 0.1% Trition-X100 in PBS for 3 min. After permeabilization cells were washed twice with wash buffer and then blocked for 30 min at room temperature in Odyssey Blocking Buffer (Thermo Fisher Scientific, Waltham, MA). After blocking anti-vinculin antibody was diluted to 1:250 in blocking buffer and incubated for 1 h at room temperature. Cells were then washed three times in wash buffer. After washing ReadyProbes AlexaFluor 488 (Thermo Fisher Scientific, Waltham, MA) was diluted in PBS according to the manufactures protocol. Samples were then incubated in the PBS solution for 1 h at room temperature and protected from light. After incubation samples were washed three times in wash buffer and then DAPI stain, diluted at 1:1,000 in PBS, was added and incubated at room temperature for 5 min. After incubation samples were mounted on glass slides using ProGold Antifade Mounting Solution (Thermo Fisher Scientific, Waltham, MA).

#### Focal Adhesion Characterization

Confocal images of the various samples were gathered using an LSM 710 Confocal Microscope (Zeiss, Jena, Germany). Briefly, a 30 μm z-stack of each sample was taken with 10 slices per stack. The image that provided the greatest resolution of vinculin (GFP^+^) was then used for further analysis and quantification. Using custom MATLAB-based software, images were analyzed for focal adhesion size and morphology, and number normalized to cell number. A contrast threshold of 0.450 was selected and applied to all images allowing segmentation of the individual focal adhesions. If the segmentation did not accurately represent the original image, a different threshold value was chosen manually until segmentation of the focal adhesions was representative of the original images. Thresholding analysis was then performed and focal adhesion number, length and width dimensions (um), and area (um^2^) were output and recorded.

#### β-Catenin Cellular Dynamics

The 30% HA/PCL substrate, which elicited the greatest increase in focal adhesion maturation and osteoblast differentiation, along with TCPS (control), were further evaluated to asses β-catenin cellular dynamics. hFOB 1.19 cells were seeded onto either TCPS or pro-osteogenic (30% HA/PCL) substrates at 100,000 cells/cm^2^ and cultured under differentiation conditions for 4, 12, 24, 48, 72, or 96 h. After culture, cells underwent protein fractionation using the Subcellular Protein Fractionation kit for Cultured Cells (Thermo Fisher Scientific, Waltham, MA). After fractionation, 10 μL of cell lysate from each fraction was removed and used to quantify total protein concentration using a Pierce BCA protein assay kit (Thermo Fisher, Waltham, MA). Twenty-five microgram of total protein from each cell fraction at the various time points was then used to quantify total β-catenin protein concentration in the various cell fractions over time using a Human Total β-catenin DuoSet IC ELISA (R&D Biosystems, Minneapolis, MN). All cell fractions were then normalized to total β-catenin concentration at the respective timepoints.

#### Luciferase Based β-Catenin Translocation Reporter Assay

To validate nuclear β-catenin translocation and activity from cell fractionation experiments, hFOB 1.19 cells were examined using a luciferase based reporter assay (Promega Corporation, Madison, WI) as previously described (Gupta et al., 2019). Briefly, hFOB1.19 cells were transiently transfected with either a TOPFLASH reporter construct, which contains a firefly luciferase reporter that is activated by TCF/LEF binding or a FOPFLASH reporter construct, which has a mutated TCF/LEF protein that prevents β-catenin activation of the firefly reporter. After 24 h, 100,000/cm^2^ cells were seeded onto either TCPS or the pro-osteogenic substrate (30%) and cultured for 48 and 72 h. After culture, the cells were lysed and a TOPFLASH/FOPFLASH quantified. β-catenin translocation and activity in cells on TCPS or the pro-osteogenic substrate was then calculated.

#### Inhibition of β-Catenin Binding to TCF/LEF

To determine if observed changes in differentiation were, in part, a result of β-catenin activity, we inhibited β-catenin bind to TCF/LEF using a small molecule inhibitor PNU-74564 (Yan et al., 2017). Under normal physiological conditions β-catenin binds TCF/LEF upon translocation to the nucleus, which then activates transcription of various genes associated with osteoblastic differentiation and other genes such as Axin2 (Leung et al., 2002; Li et al., 2018). Upon introduction of PNU-74564, the binding of β-catenin is inhibited, thus inhibiting downstream effector gene expression. hFOB 1.19 cells were seeded onto either TCPS or pro-osteogenic (30% HA/PCL) at 100,000/cm^2^. The cells were then exposed to either differentiation media supplemented with 0.1% dimethyl sulfoxide (DMSO) or differentiation media supplemented with 0.1% DMSO and 25 μM PNU-74564. Cells were then cultured under differentiation conditions for 2, 3, or 7 days with media changed every 2 days. At each time point cells were evaluated for AP activity as described previously in section Alkaline Phosphatase (AP) Activity, or for Axin2 gene expression using methods described previously in section Osteoblastic Gene Expression. Glyceraldehyde-3-Phosphate Dehydrogenase (*Gapdh*) was used as a reference gene for all samples. All primers used were PrimePCR Sybr Green Assay Primers (Bio-Rad Laboratories, Hercules, CA). The ΔΔCt methodology was used as described previously to compared changes in gene expression relative to Axin2 gene expression in the control cells cultured on TCPS at day 2 (Rao et al., 2013).

### Statistical Evaluation

Changes in substrate surface properties were assessed with 3 or more samples with each sample being the average of three replicates. Gene expression was assessed with 3–4 samples with each sample being the average of three replicates. Focal adhesion quantification and characterization was assessed with 3 samples with each sample consisting of ~5–75 cells or ~500–5,000 focal adhesions per sample. β-catenin localization was assessed with 4–6 samples with each sample consisting of two replicates. Osteoblastic gene expression and differentiation in response to β-catenin inhibitor PNU-74564 was assessed with 5–6 samples with each sample being the average of 3 replicates or 2 replicates for gene expression and AP activity, respectively. Changes in surface properties and osteoblastic AP activity were assessed using two-way ANOVA followed by Tukey's *post-hoc* analysis and are reported as mean ± SD. Changes in osteoblastic gene expression and focal adhesion size and eccentricity were assessed using two-way ANOVA followed by Tukey's *post-hoc* analysis and are reported as mean ± SD. Changes in β-catenin localization were assessed using two-way ANOVA followed by Tukey's *post-hoc* analysis and are reported as mean ± SD. Changes in β-catenin translocation and activity, determined using a luciferase-based reporter assay, was assessed using an unpaired *t*-test followed by Bonferroni *post-hoc* analysis and are reported as mean ± SD. The effect of β-catenin inhibitor, PNU-74564, on Axin2 gene expression and AP activity were assessed using two-way ANOVA followed by Tukey's *post-hoc* analysis and are reported as mean ± SD. All analysis was performed using GraphPad Prism version 8.1.1 for Windows (GraphPad Software, La Jolla California USA). *P* < 0.05 were considered statistically significant.

## Results

### Substrate Surface Roughness and Particle Density Varied Based on HA Concentration

Characterization of the roughness, substrate height, particle density, and wettability for the glass substrate, PCL substrate, 5% HA/PCL, 30% HA/PCL, and 50% HA/PCL substrates is summarized in Table 2. The substrate height of glass and PCL only substrates did not vary significantly from each other with observed values of 543 ± 8 nm and 598 ± 82 nm, respectively. However, these values were significantly different than all other substrates evaluated. No significant variation in substrate height was observed between the 5 and 30% substrates with observed heights of 1,063 ± 229 nm and 969 ± 361 nm, respectively, while the 50% substrate with an average height of 2,165 ± 121 was significantly different than all other substrates evaluated. Substrate wettability did not vary significantly between any of the evaluated substrates, but surface roughness of the 50% HA/PCL substrate was greater than all other substrates. Particle density did vary significantly between the Glass, TCPS, and 5% substrates with particles densities of 0 ± 0, 3 ± 1, and 16 ± 2 p/cm, respectively, compared to the 30 and 50% substrates with particle densities of 86 ± 2 and 165 ± 8 p/cm, respectively. The 30 and 50% substrates were significantly different than all other substrates and significantly different from each other.

**Table 2:** Substrate characteristic characterization and evaluation of osteoblastic differentiation.

**Substrate**	**Height (nm)**	**Roughness (Ra)**	**Surface energy (°)**	**Particle density (particles/cm)**	**AP activity (U/μg)**
TCPS	–	–	–	–	13 ± 4.9
Glass	543 ± 8^a^	2 ± 2.19	84.32 ± 1.11	0 ± 0^a^	13 ± 5.4
PCL	598 ± 82^a^	8 ± 19.16	85.88 ± 1.11	3 ± 1^a^	13 ± 6.4
5%	1063 ± 229^b^	21 ± 9.58	88.24 ± 0.73	16 ± 2^a^	17 ± 9.8
30%	969 ± 361^b^	25 ± 7.97	87.42 ± 0.98	86 ± 2^b^	32 ± 9.2^a^
50%	2165 ± 121	94 ± 3.53^a^	92.03 ± 0.36	165 ± 8^c^	15 ± 9.9

*n ≥ 3 with each sample being the average of 3 replicates. Groups sharing the same letter are not significantly different (p < 0.05) within the same column*.

### Osteoblastic Differentiation Is Increased On 30% HA/PCL Substrate

**Figure 1:**
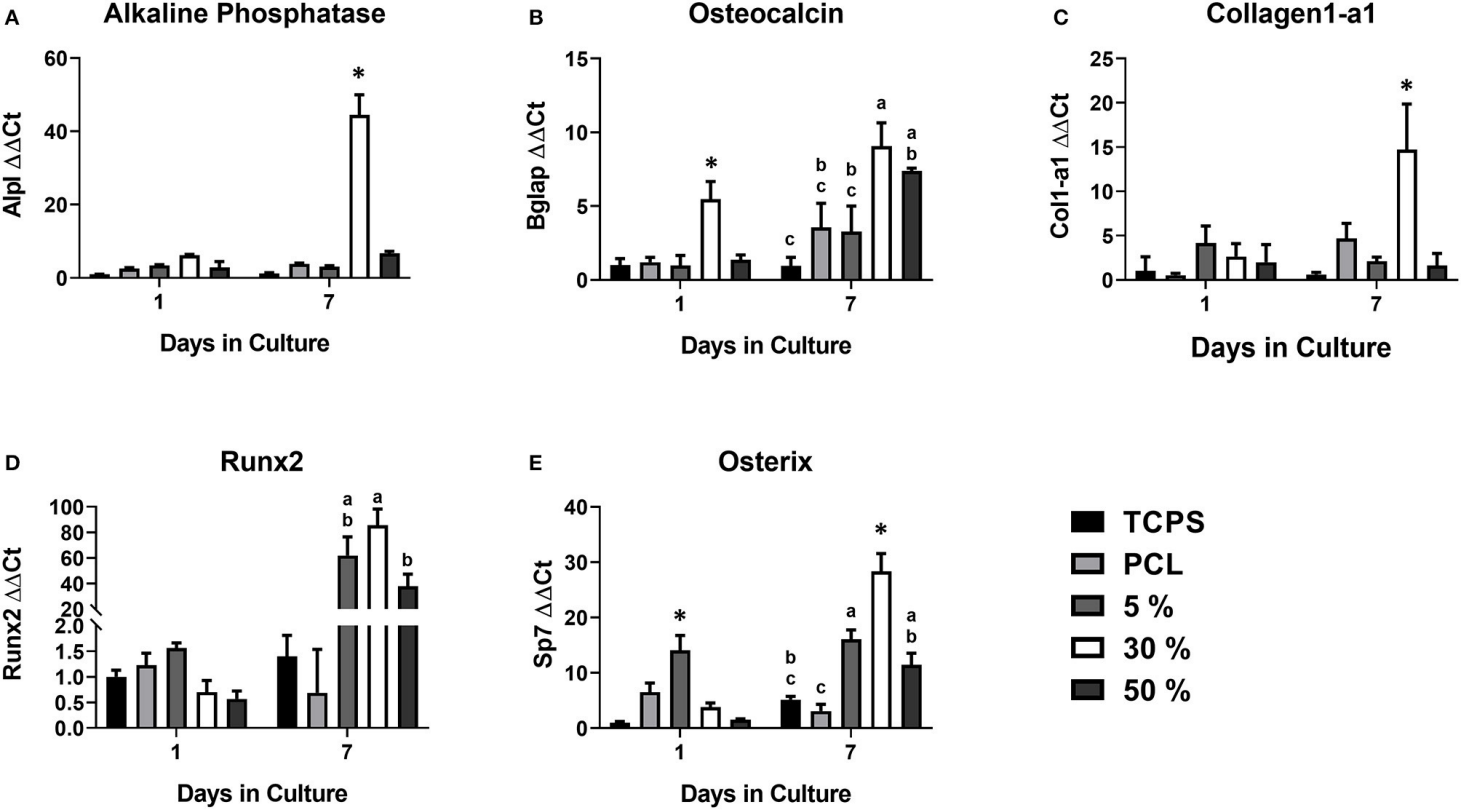
Osteoblastic gene expression on the various PCL/HA substrates. Evaluation of osteoblastic gene expression of **(A)** alkaline phosphatase (Alpl), **(B)** osteocalcin (Bglap), **(C)** collagen1-a1 (Col1-a1), **(D)** Runx2, and **(E)** Osterix (Sp7) at 1 and 7 days on the various substrates evaluated in this study. Substrates sharing the same letter denotes a lack of significance at the same time point, *significantly different compared to all other groups at the same time point. *n* = 3–4 samples with each sample being the average of three replicates.

In order to assess osteogenic potential of the various substrates, expression of key osteoblastic differentiation markers were assessed at days 1 and 7 (Figure 1). At day 1, Bglap expression was significantly upregulated in cells on the 30% substrates vs. all other substrates. Sp7 expression was significantly upregulate in cells on the 5% substrate compared to all other substrates, however these results varied based on HA/PCL composition. Trends were more apparent after 7 days of culture. Runx2 gene expression, a marker of commitment to the osteoblast lineage, was significantly increased in cells on substrates that contained HA (5, 30, and 50%) compared to cells that were seeded on substrates that did not contain HA (TCPS, PCL). This suggests HA has a significant impact on osteoblastic differentiation, regardless of the substrate characteristics. Cells on the 30% HA/PCL substrate had significantly greater gene expression in genes related to bone matrix deposition (*Alpl, Col1a1*, and *Bglap*) and *Sp7* than all other groups. This indicated that the 30% HA/PCL substrate promotes greater osteoblastic differentiation compared to other substrates. To confirm the results observed at day 7 on the various substrates, AP activity was quantified for cells on each substrate. After 7 days of culture cells on the 30% substrate exhibited a significant 1-fold increase in AP activity compared to cells on any of the other substrates (Table 2). Significant variations in AP activity was not observed in cells on any of the other substrates.

### Osteoblast Focal Adhesion Maturation Was Increased by 30% HA/PCL Substrate

**Figure 2:**
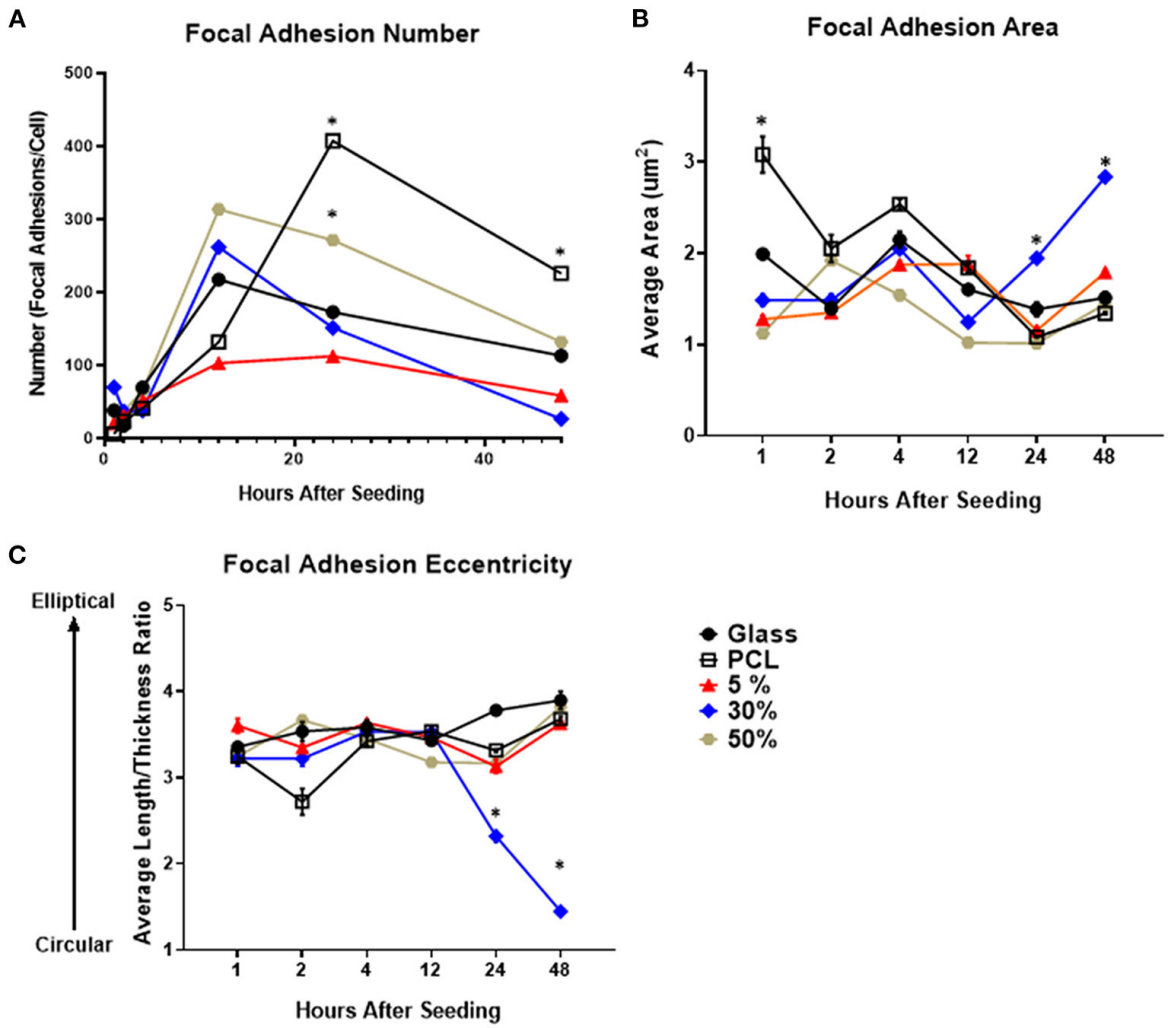
Focal adhesion number, size, and morphology over time on each of the evaluated substrates. Temporal quantification of focal adhesion **(A)** number, **(B)** area, and **(C)** eccentricity over time on either glass, PCL, 5, 30, or 50% substrates. *p < 0.05 compared to all other groups at the same time point. *n* = 3 samples with each sample consisting of ~5–75 cells or ~500–5,000 focal adhesions per sample.

The number of focal adhesions per cell was evaluated for all substrates (Figure 2A). Over the first 12 h all substrates demonstrated a dramatic increase in focal adhesion number. After 24 h the number of adhesions per cell begins to decrease on all substrates aside from the PCL substrate. From 12 to 24 h, the 30% substrate had the greatest decrease in adhesion number, decreasing by roughly 100 adhesions per cell. Conversely, the total number of adhesions per cell on glass, 5% HA/PCL and 50% HA/PCL substrates decreased by roughly 50 adhesions per cell. The decrease in adhesion number continued at 48 h when the evaluation concluded. Again the 30% HA/PCL substrate had the greatest decrease in adhesion number per cell compared to the glass, 5 and 50% HA/PCL substrates. The number of adhesions per cell in cells on the PCL substrate did decrease drastically between 24 and 48 h but remained higher than all other substrates.

To further evaluate focal adhesion maturation, both adhesion size and eccentricity were quantified. The average area of focal adhesions in cells on the 30% HA/PCL substrate was significantly increased 24 and 48 h after cell seeding compared to all other substrates (Figure 2B). Furthermore, compared to cells on all other substrates, a 1- and 3-fold increase in adhesion eccentricity (value closer to 1) was observed in cells on the 30% HA/PCL substrate 24 and 48 h, respectively, after cell seeding. We also observed differences in focal adhesion morphology in cells cultured on other substrates 2 h after cell seeding (Figure 2C). These variations in adhesion morphology were not as much as those observed 24 and 48 h after cell seeding on the 30% substrate and therefore are not discussed in detail. The increase in focal adhesion area and decrease in overall number are typical markers of focal adhesion maturation (Biggs and Dalby, 2010). These results indicate that the 30% HA/PCL substrate caused more rapid focal adhesion turnover and maturation compared to all other substrates evaluated.

### More Rapid β-Catenin Protein Translocation From Cell Membrane to Nucleus on Pro-Osteogenic Substrate (30% PCL/HA)

**Figure 3:**
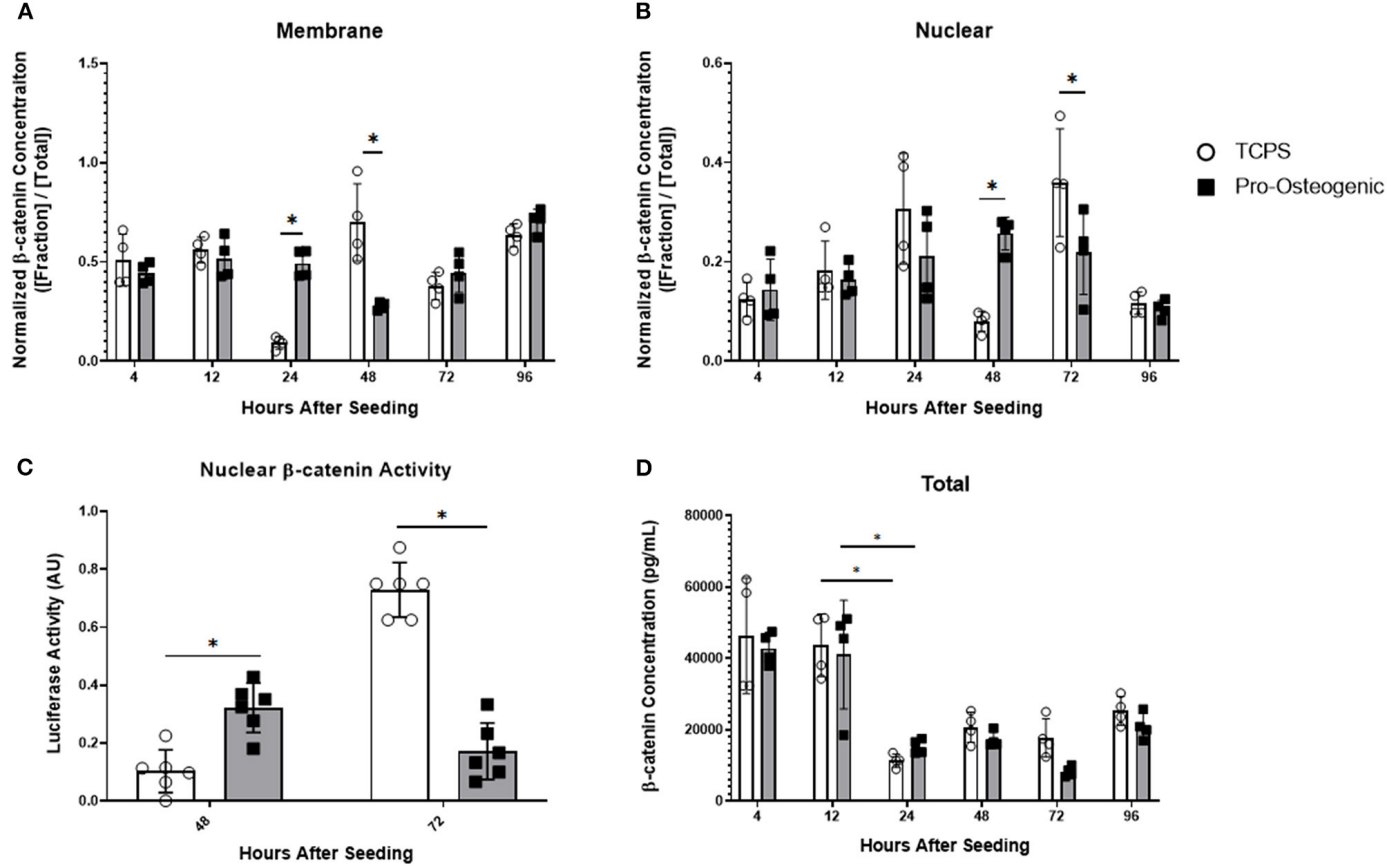
β-catenin localization and total β-catenin concentration over 96 h and transcriptional activity at 48 and 72 h in hFOB 1.19 cells cultured on either TCPS or pro-osteogenic substrate. Quantification of normalized β-catenin concentration in either the **(A)** membrane bound cell fraction, **(B)** nuclear cell fraction, or **(D)** total β-catenin concentration. **(C)** Nuclear β-catenin activity quantified by TOPFLASH activity on either TCPS or pro-osteogenic substrate after 48 h and 72 h. *n* = 4–6 samples with each sample consisting of two replicates. *p < 0.05.

To further examine our hypothesis, we examined sub-cellular β-catenin localization with protein fractionation and ELISA to quantify β-catenin localization within the cell. We also evaluated transcriptional activity using a TOPFLASH reporter assay (Figure 3). Because we sought to evaluate the mechanism by which substrate surface characteristics induce osteoblastic differentiation, and because the 30% HA/PCL substrate was the only substrate that induced osteoblastic differentiation (Table 2 and Figure 1), it was the only substrate that was evaluated in further detail. We will refer to the 30% substrate as the “pro-osteogenic substrate” henceforth because it enhanced pro-osteogenic capacity and induced greater focal adhesion maturation. Data from cells cultured on TCPS is presented as a reference to more clearly illustrate the observed variations of β-catenin localization and activity in cells cultured on the 30% substrate which have undergone significant differentiation compared to cells cultured on a substrate that did not induce differentiation. At all time points, cytoplasmic, membrane, nuclear, and cytoskeletal fractions were isolated and normalized to total β-catenin concentration at the respective timepoint. β-catenin concentration in the cytoplasmic and cytoskeletal fraction was not significantly different at any time point and is therefore not shown. At 4, 12, and 96 h after seeding cells onto either TCPS or pro-osteogenic substrate, no cell fraction exhibited significant differences in β-catenin concentration. Twenty-four hours after cell seeding, no significant variations in total β-catenin concentration in the nuclear fraction was observed between cells cultured on TCPS or pro-osteogenic substrate. However, in the membrane bound fraction, where β-catenin is localized at adherens junctions or focal adhesions, we observed a significant increase in β-catenin concentration in cells on pro-osteogenic substrates compared to cells on TCPS (Figure 3A). At 48 h, in the membrane bound fraction we observed a significant increase in β-catenin concentration in cells on TCPS compared to cells on the pro-osteogenic substrate (Figure 3A). Cells on pro-osteogenic substrates also displayed a significant increase in β-catenin concentration within the nuclear fraction compared to cells cultured on TCPS (Figure 3B). At 72 h, no significant variations in normalized β-catenin concentration within the membrane bound fraction was observed between cells on TCPS and cells on the pro-osteogenic substrate. However, there was a significant increase in normalized β-catenin concentration in the nuclear fraction of cells on TCPS compared to cells on pro-osteogenic substrate. To confirm these findings we used a luciferase-based TOPFLASH reporter assay that indicates increased transcriptional activity of TCF/LEF caused by β-catenin binding within the nucleus (Figure 3C). We observed a significant increase in TOPFLASH activity in cells on the pro-osteogenic substrate compared to cells on TCPS at 48 h. At 72 h we observed a significant increase in TOPFLASH activity in cells on TCPS compared to cells on pro-osteogenic substrate. Both of these results corroborate our finding as regards the translocation of β-catenin observed when using protein fractionation coupled with ELISA. Total cellular β-catenin was also quantified and no significant difference in cells on TCPS compared to cells on pro-osteogenic substrate at any of the examined time points (Figure 3D). Interestingly, at 4 and 12 h the total, concentration of β-catenin, i.e., combining membrane and nuclear fractions, was roughly 4,500 pg/mL. At 24 h the total concentration of β-catenin within the cells decreased almost 1.5-fold, to ~1,500 pg/mL, in cells cultured on both the TCPS and pro-osteogenic substrate. The total β-catenin concentration within the cells cultured on TCPS and pro-osteogenic substrate then remained at the lower concentration for the remainder of the study.

### Inhibition of β-Catenin Binding to TCF/LEF Prevents Substrate-Induced Increases in Osteoblastic Differentiation

**Figure 4:**
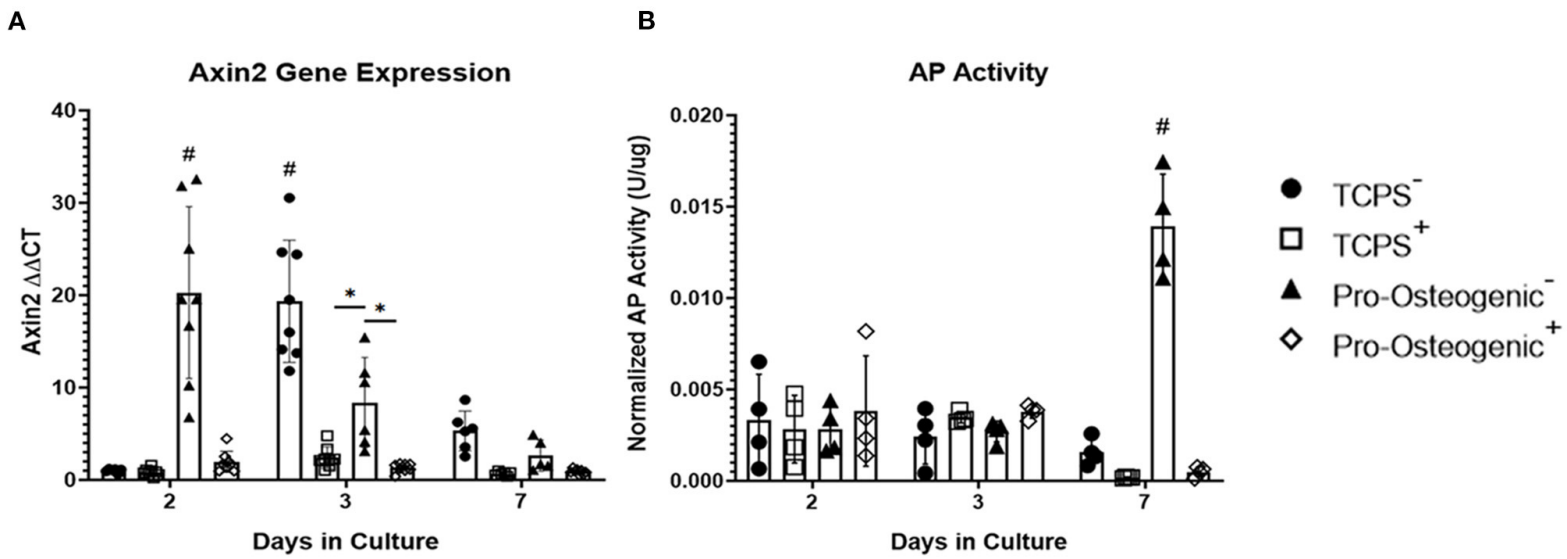
Inhibition of β-catenin binding to TCF/LEF in hFOB 1.19 cells on TCPS or Pro-Osteogenic Substrates. Evaluation of effect of β-catenin inhibitor, PNU-74564, on **(A)** Axin2 gene expression and **(B)** AP Activity in cells cultured in media with inhibitor (+) or without inhibitor (–) on either TCPS or Pro-Osteogenic substrates. *p < 0.05, ^#^*p* < 0.05 compared to all other groups at same time point. *n* = 5–6 samples with each sample being the average of 3 replicates or 2 replicates for gene expression and AP activity, respectively.

To examine whether the observed changes in osteoblastic differentiation were, in part, a result of the observed increase in β-catenin translocation to the nucleus we then inhibited β-catenin's ability to bind to TCF/LEF using PNU-74564 (Yan et al., 2017). We examined the inhibitor's efficacy by evaluating Axin2, a downstream effector gene of TCF/LEF (Leung et al., 2002). After 2 days in culture, in the absence of PNU-74564, Axin2 gene expression increased significantly in cells cultured on pro-osteogenic substrates compared to cells cultured on TCPS (Figure 4A). However, the observed increase in Axin2 expression in cells on pro-osteogenic substrates was inhibited in the presence of PNU-74564. After 3 days in culture, cells cultured on either TCPS or pro-osteogenic substrate, in the absence of PNU-74564, displayed a significant increase in Axin2 gene expression compared to cells cultured on the same substrate in the presence of PNU-74564. Moreover, in the absence of PNU-74564, Axin2 gene expression in cells cultured on TCPS was significantly increased compared to cells cultured in the absences of PNU-74564 on pro-osteogenic substrate. After 7 days in culture, no significant changes in Axin2 gene expression was observed in any of the groups evaluated. AP activity was significantly increased after 7 days in cells cultured on pro-osteogenic substrate not in the presence of PNU-74564. This increase was inhibited in cells exposed to PNU-74564 (Figure 4B).

## Discussion

Development of substrate surface characteristics to promote osteointegration has been an area of intense interest for decades. However, the specific mechanism by which substrate surface characteristics are transduced, as well as what substrate characteristic may regulate this mechanism, are still largely unknown. We hypothesized that the 30% HAp particle density may be a key regulator of osteogenesis. Further, liberation of β-catenin from focal adhesions as they undergo maturation and the subsequent translocation of β-catenin to the nucleus is a possible mechanism by which substrate surface characteristics are transduced. We observed that only one of the evaluated substrates, the 30% PCL/HA substrate, induced an increase in osteogenic gene expression, AP activity and focal adhesion maturation (Figure 1). We also determined that the cells on the 30% PCL/HA substrate had more rapid β-catenin translocation from the cell membrane to the nucleus than cells on TCPS. These results suggest that focal adhesion turnover, by releasing β-catenin, increases cellular osteogenic activity by increasing nuclear β-catenin transcriptional activation.

Upon evaluation of the 30% HAp substrate surface characteristics, only particle density differed significantly between all other substrates examined in this study. Substrate height, roughness, and wettability were not significantly different between the substrates examined (Table 2). However, literature suggests that the determination of which substrate surface characteristics regulate osteogenesis is highly complex.

Investigation by Dalby et al. and Khang et al. determined that substratum roughness and height directly affected osteoblastic response (Dalby et al., 2002; Khang et al., 2012), a finding not corroborated by our study. Specifically, they determined that micron scale topographies with a substrate height between 100–300 nm and nanoscale roughness between 12 and 14 nm elicited increased osteoblastic differentiation. Lim et al. proposed that substratum surface height was responsible for inducing osteoblastic differentiation (Lim et al., 2012). They determined that isotropic topography with a height of 11 nm induced greater osteoblastic differentiation, proliferation, focal adhesion formation and cell spreading compared to 35 and 85 nm high topographies *in vitro* (Lim et al., 2012). A study by Loiselle et al. evaluated substratum topography *in vitro* and in *vivo* (Loiselle et al., 2013), with findings contradictory to those observed by Lim et al. Loiselle et al. observed that 50–60 nm substratum height induced the greatest osteoblastic differentiation *in vitro* as well as greater *in vivo* mineralization compared to 10–15 nm and 45 nm substratum height (Loiselle et al., 2013). In the study presented here we found no significant differences in surface roughness between the 5, 30, and 50% substrates, with roughness averages of 21, 25, and 94, respectively. Although this study evaluated significantly larger substratum surface heights (0.5–2 μm), variations in substratum surface height also varied independently of osteoblastic differentiation. These conflicting results suggests more investigation is needed to determine conclusively if substrate roughness and height significantly contribute and regulate osteogenesis.

Similar to substrate roughness and height, studies examining substrate wettability have yielded conflicting results. Although a correlation between substrate wettability and osteoblastic differentiation has been reported, the osteoblastic response observed has been inconsistent and varied depending on surface topography, chemistry, and the specific cell line being evaluated (Lim et al., 2005b; Lim and Donahue, 2007; Park et al., 2012). In the study presented here, surface wettability was not significantly altered between any of the substrates examined suggesting substrate wettability may not have as significant a role in regulating osteoblastic differentiation as previous studies have suggested (Lim et al., 2005b; Park et al., 2012).

Particle density, unlike the other characteristics examined in this study, was significantly different on the 30% substrate compared to the other substrates examined. The 30% substrate had a particle density of 86 particles per centimeter, roughly 70 particles per centimeter greater than the 5% substrate and 80 less particles per centimeter than the 50% substrates, which had particle densities of 16 and 165 particles per centimeter, respectively. A study by Ahn et al. examined how nanopost density influenced osteogenic commitment of human mesenchymal stem cells (hMSCs) and observed a parabolic correlation between post-density and osteogenic differentiation (Ahn et al., 2014), similar to the response observed in this study. It was determined that lower and higher nanopost densities did not induce a sufficient amount of cell spreading to increase cell stiffness and induce osteogenic differentiation. Another study by Kuo et al. corroborated these findings. They determined that 500–700 nm nanopillars reduced apoptosis and increased focal adhesion formation to the greatest extent. They also determined that roughly 900 nm between pillars was the ideal spacing to induce cell spreading (Wen Kuo et al., 2014). Our findings, coupled with the previously mentioned studies, suggest that there is an optimal density, when using roughly 500 nm topographic features that induces an increase in osteogenesis. However, the results observed in this study and others suggest that the relationship between substrate characteristics and osteogenesis is highly complex. It depends heavily on the specific materials, surface characteristics, cell type, and culture conditions being used and further study is required before definitive assertions can be made as to which substrate characteristic is responsible for inducing osteogenesis.

Interestingly, in the previously mentioned studies that examined substrate characteristics there was a commonality, despite the conflicting results as to which substrate characteristic induces osteogenesis. The substrate that resulted in the greatest increase in osteoblastic differentiation also induced an increase in focal adhesion formation and maturation (Lim et al., 2007; Loiselle et al., 2013). In a proliferative state, cells exhibit numerous, small, oblong focal adhesions. These adhesions probe the surrounding environment to evaluate the substrate. As the focal adhesions mature, they become larger and decrease in overall number. Upon investigation of the 30% HAp substrate, we observed similar changes that suggest a maturation of focal adhesions (Figure 2). This maturation was observed on the 30% substrate in conjunction with upregulation of key osteoblastic differentiation genes as well as increased AP activity, suggesting a relationship between focal adhesion maturation and osteoblastic differentiation. This suggests that while substrate surface characteristics may be variable, the substrates ability to induce focal adhesion formation and maturation may be a key factor in regulating osteoblastic differentiation. Evidence of this interaction has been observed in previous studies examining cell adhesion complexes and osteogenesis. However, these studies have failed to identify a mechanism by which focal adhesion maturation may contribute to the regulation of osteoblastic differentiation (Alenghat and Ingber, 2002; Galli et al., 2012; Abagnale et al., 2015). This unknown question has been succinctly summarized in a review by Jamora and Fuchs (2002). The question is as follows: it is known that disassociation of adhesion complexes occurs in response to various stimuli, but what happens to the proteins, such as β-catenin, that make up the adhesion complexes after disassociation? Do they participate in other facets of cell mechanotransduction, or are they broken down and recycled within the cell?

To probe the question posed by Jamora and Funches, we examined β-catenin sub-cellular localization over time. Interestingly on the pro-osteogenic substrate, β-catenin concentration remained high in the membrane after 24 h before decreasing at 48 h then returning to a concentration similar to that observed over the first 12 h. At 48 h, β-catenin concentration in cells on the pro-osteogenic substrate exhibited an increase in nuclear translocation of β-catenin, which coincided with the decrease in β-catenin at the membrane. After 48 h nuclear β-catenin concentration returned to levels observed over the first 24 h. Cells on TCPS exhibited the same shift of β-catenin from the membrane to the nucleus and then back to basal levels over a 24-h period, similar to that observed on the pro-osteogenic substrate. However, unlike cells on the pro-osteogenic substrate, in cells on TCPS the shift of β-catenin from the membrane to the nucleus did not begin until 48 h after seeding, 24 h later than the pro-osteogenic substrate (Figure 3). This more rapid translocation of β-catenin in cells on pro-osteogenic substrate is likely due to one of the three mechanism discussed previously. The increase in nuclear localization of β-catenin at 48 h on the pro-osteogenic substrate also coincided with an increase in TOPFLASH activity indicating an increase in TCF/LEF transcriptional activity, which is pivotal in the regulation of osteogenesis (Figure 3D). It is also worth noting that Axin2 gene expression closely mirrors the transcriptional activity of β-catenin as measured by the TOPFLASH reporter assay, further corroborating the observed changes (Figure 4A).

We further confirmed that the observed increase in AP activity on the pro-osteogenic substrate is, in part, a result of the more rapid translocation of β-catenin to the nucleus as well as the observed increase in transcriptional activity. Inhibiting β-catenin binding to TCF/LEF inhibits upregulation of downstream effector genes, many of which regulate osteoblastic differentiation (Li et al., 2018). Upon inhibition using PNU-74564, a significant decrease in Axin2 gene expression was observed in cells cultured on pro-osteogenic substrates and TCPS after 2 and 3 days, respectively (Figure 4A). Importantly, the significant increase in AP activity that is typically associated with the pro-osteogenic substrate was inhibited in cells cultured in the presence of PNU-74564 (Figure 4B). This suggests that the observed increase in AP activity is a consequence of the more rapid β-catenin translocation and increase in transcriptional activity. Taken together these results support our hypothesis that the osteogenic substrate we identified in this study induces the liberation of β-catenin from adhesion complexes allowing β-catenin to translocate to the nucleus and activate osteoblastic differentiation. This also provides further evidence that β-catenin bound at adhesion complexes and β-catenin that actively participates in intracellular signaling come from the same pool, a concept suggested by results from previous studies (Nelson and Nusse, 2004; Bienz, 2005; Mbalaviele et al., 2006).

We hypothesized that β-catenin, a protein bound at the cell adhesion complexes, is liberated and is then free to translocate to the nucleus where it can bind TCF/LEF and regulate osteogenic gene expression. Evidence from the study presented here, as well as previous studies, suggests one of three mechanism by which focal adhesions may mediate the observed change in β-catenin localization and activity. The first is that as maturation of focal adhesions occurs, greater intracellular tension is generated. Increasing tensile force within the cells then causes disassociation of cadherin complexes therefore liberating β-catenin. This concept is supported by previous studies that have observed increased breakdown and turnover of E-cadherins in response to increased contractile activity (Kale et al., 2018). The second is that β-catenin may participate in the binding of vinculin to actin filaments at focal adhesions similar to that observed at adherens junctions. As focal adhesion turnover occurs, β-catenin can then be liberated from the focal adhesion complexes. Recent studies suggest β-catenin interacts with paxillin during increased Rac activation, which occurs during focal adhesion turnover (Mackay et al., 1997; Birukova et al., 2007; Spiering and Hodgson, 2011; Dubrovskyi et al., 2013). In addition, it is reasonable to suggest that β-catenin binds vinculin at focal adhesions in a manner similar to what is observed at adherens junctions, and then as turnover occurs it is liberated from the adhesion complex (Peng et al., 2010). The third possibility is that while β-catenin may assist in focal adhesion formation and stabilization, and therefore localize at these locations, focal adhesions may mediate β-catenin translocation through a secondary messenger. One proposed mechanism is that focal adhesion phosphorylation, which occurs during adhesion turnover, causes Akt phosphorylation which then inhibits GSK-3β, a protein that phosphorylates β-catenin. The decreased β-catenin phosphorylation leads to an increase in cytosolic β-catenin and, in turn increased β-catenin nuclear translocation and activity (Case et al., 2011; Thompson et al., 2012). However, this mechanism has only been examined in mesenchymal stem cells with respect to adipogenesis. It has not been evaluated in osteoblastic cells or with respect to osteogenesis.

Further examination of the interplay between focal adhesion maturation, β-catenin shuttling, and osteoblastic differentiation is required to evaluate our hypothesized mechanism. Wnt signaling is known to be regulated by various stimuli and the study presented here does not encompass or evaluate the wide array of upstream and downstream Wnt targets such as Akt phosphorylation (Hill et al., 2005; Case et al., 2011; Baron and Kneissel, 2013; Thompson et al., 2013). The studies discussed previously have observed noticeable changes in focal adhesion size and shape on pro-osteogenic substrates (Lim et al., 2005a, 2007). Unfortunately, the focal adhesions were not characterized fully for maturation and therefore do not allow for a determination to be made on the relationship between focal adhesion maturation and osteogenesis. Lastly, inhibition of this pathway further upstream, ideally at the focal adhesion complex, would provide greater insight into the mechanism by which β-catenin is liberated from focal adhesions. However, inhibitors that more directly interfere with β-catenin signaling or the focal adhesions cause significant phenotypic changes regardless of the substrate they are being cultured on, making experimental results difficult to interpret (Hill et al., 2005; Prowse et al., 2013; Rutkovskiy et al., 2016).

## Conclusion

This study examined the role of substrate surface characteristics to determine if a singular characteristic was regulating osteoblastic differentiation. Additionally, this study examined the correlation between osteoblastic differentiation, focal adhesion formation and maturation, and suggested and evaluated a novel mechanism to explain this correlation. We found that one substrate, the pro-osteogenic 30% HA/PCL substrate, increased AP activity and expression of genes associated with osteoblastic differentiation as a result of a more rapid translocation and increase in β-catenin transcriptional activity. We concluded that in this study only particle density, and therefore the distance between topographic features, was significantly different. Thus our findings, in addition to information from previous studies (Wen Kuo et al., 2014; Di Cio and Gautrot, 2016), suggest that particle density is possibly one substrate characteristic which regulates osteogenic potential. However, more study is needed as the relationship between substrates and osteogenesis is complex and depends on various factors such as cell type and substrate materials.

Cells on the 30% substrate also exhibited variations in focal adhesion formation and morphology, becoming larger and more eccentric compared to cells on all other substrates. These observations are indicative of focal adhesion turnover and maturation and similar to those seen on other pro-osteogenic substrates. Additionally, we found that there is a translocation of β-catenin from the membrane fraction at 24 h to the nuclear fraction at 48 h on pro-osteogenic substrate, 24 h earlier than that which was observed in cells on TCPS. Furthermore, β-catenin was not only more localized to the nucleus on 30% HA/PCL, it was also bound TCF/LEF more frequently, indicating increased β-catenin transcriptional activity. It was further determined that by inhibiting β-catenin binding of TCF/LEF, the increase in osteoblastic differentiation typically observed in cells cultured on the pro-osteogenic substrate was inhibited. Taken together these results suggest that as focal adhesions mature, reducing in number in response to topography, the liberated β-catenin is then translocated to the nucleus and actively binds TCF/LEF. This would indicate that a focal adhesion initiated β-catenin mediated mechanism contributes to the transduction of substrate surface characteristics. More study is required to determine which of the three possible mechanisms described previously of β-catenin and focal adhesion interaction are occurring and causing the observed β-catenin translocation and enhanced osteogenic response.

## Data Availability Statement

The raw data supporting the conclusions of this article will be made available by the authors, without undue reservation.

## Author Contributions

OJ and A-BM performed experimental research, wrote, and proofread the manuscript. YZ provided guidance and assistance on multiple experimental protocols as well as data anlaysis and manuscript revision. CL provided guidance on focal adhesion mechanics, staining, and the software for measurements in addition to proofreading of the manuscript. HD provided funding for the research, proofreading of the manuscript, and guidance on experimental protocols. All authors contributed to the article and approved the submitted version.

## Conflict of Interest

The authors declare that the research was conducted in the absence of any commercial or financial relationships that could be construed as a potential conflict of interest.
