# Supplementary material for: Hydroxyapatite Particle Density Regulates Osteoblastic Differentiation Through β-Catenin Translocation
Source: Front Bioeng Biotechnol. 2021 Jan 8;8:591084. doi: 10.3389/fbioe.2020.591084 (PMC7820766; doi:10.3389/fbioe.2020.591084)
Supplement: Supplementary file 1 [file Data_Sheet_1.PDF]

Supporting Information for Publication

Hydroxyapatite Particle Density Regulates Osteoblastic Differentiation Through β-catenin Translocation

Otto Juhl IV[1], Anna-Blessing Merife[1], Yue Zhang[1], Christopher Lemmon[1], Henry J. Donahue[1*]

[1]Department of Biomedical Engineering and Institute for Engineering and Medicine, Virginia Commonwealth University, Richmond VA, 23220

***Corresponding Author: hjdonahue@vcu.edu**

Six pages and one figure of supporting information for publication.

Page S1-S6... *Figure S1*

GLASS SUBSTRATE

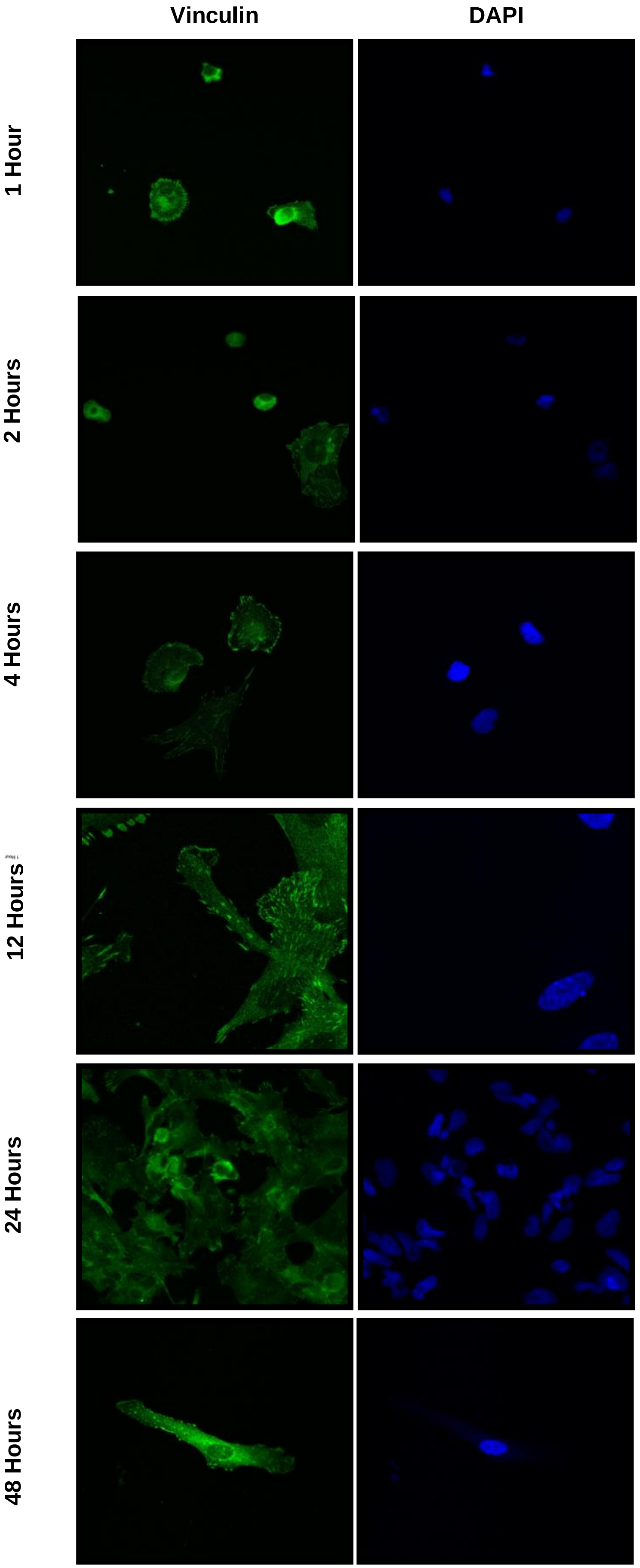

PCL SUBSTRATE

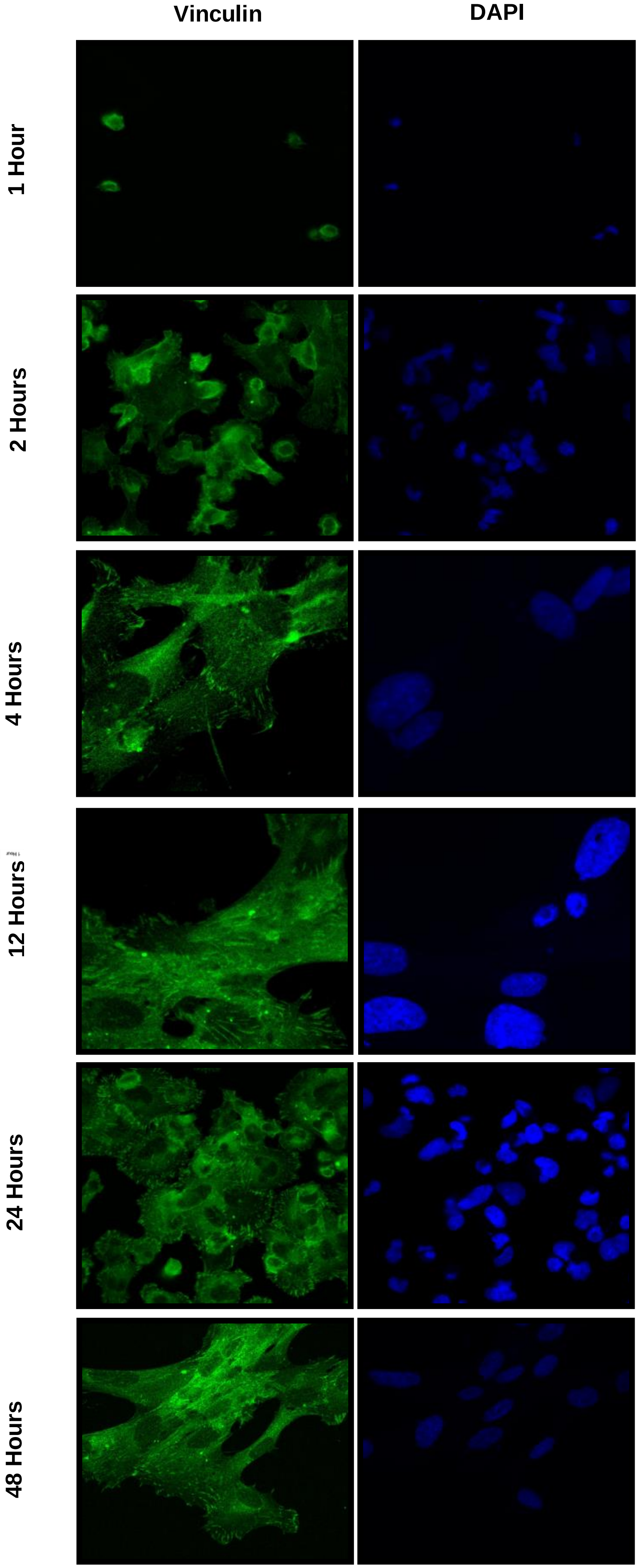

5% HA/PCL SUBSTRATE

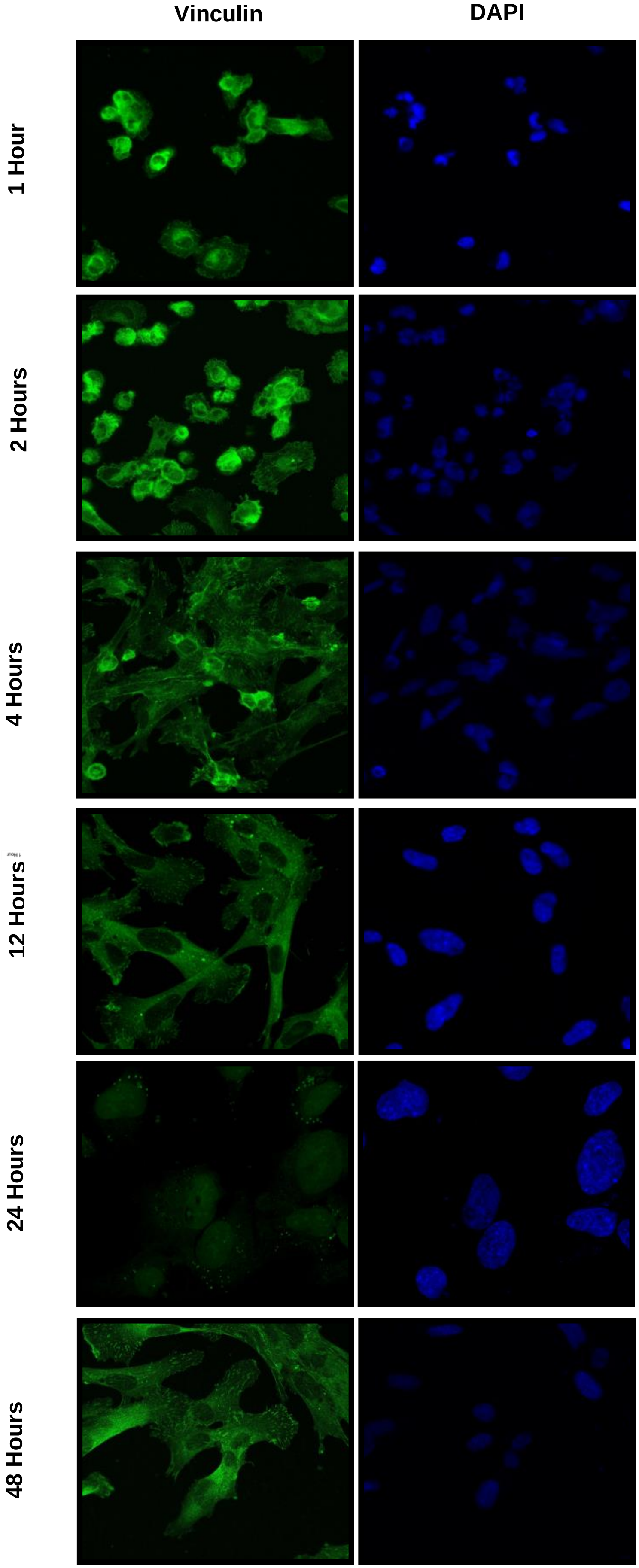

30% HA/PCL SUBSTRATE

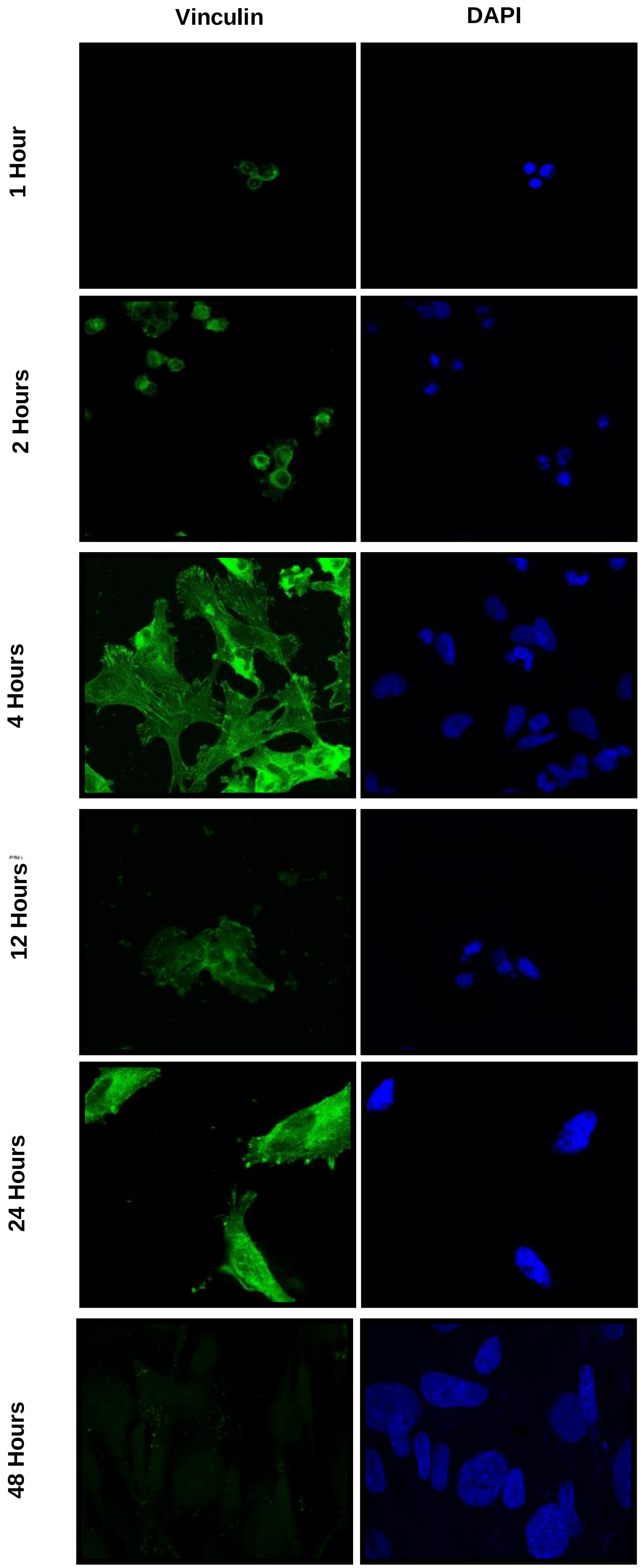

50% HA/PCL SUBSTRATE

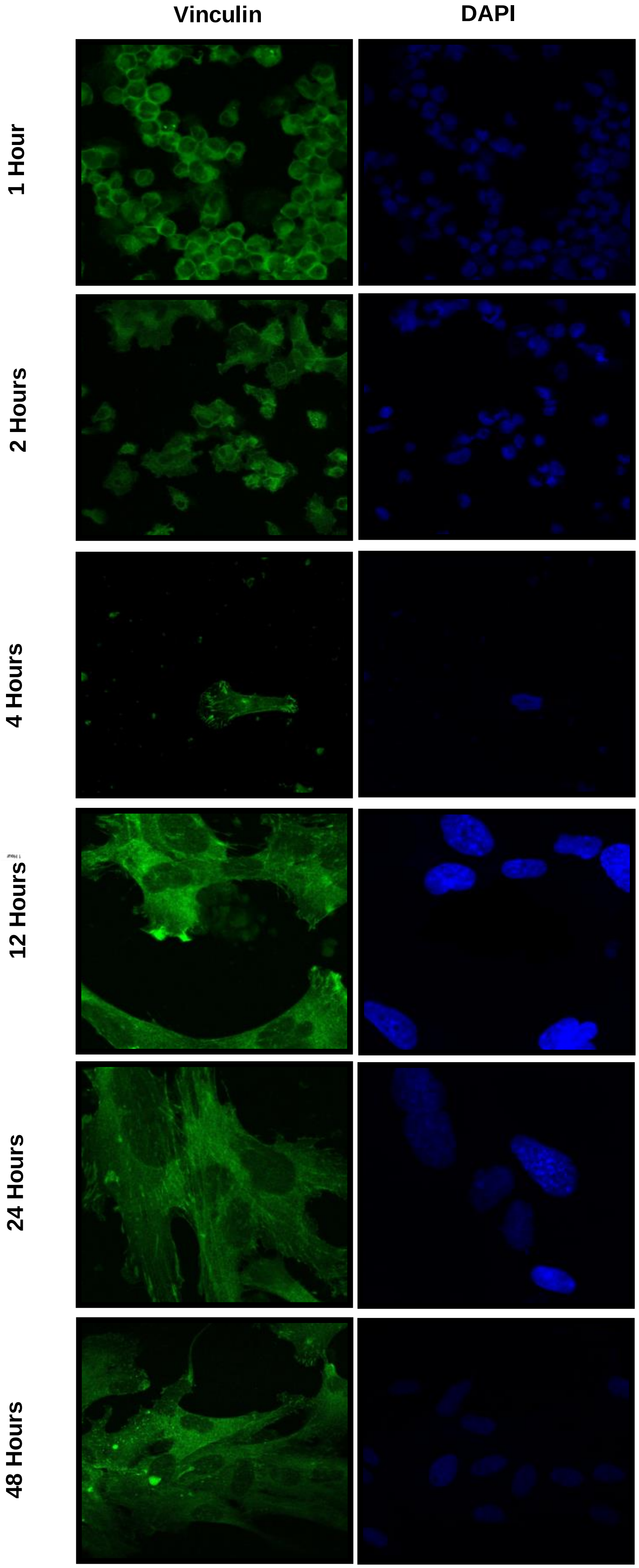

Figure S1. Fluorescent staining of focal adhesion complexes (vinculin) and the nucleus (DAPI) in cells cultured on Glass, PCL, 5%, 30% or 50% substrates over 48 hours.
